# Analysing the loss of embryogenic competence in long-term cell lines of *Solanum betaceum* Cav.: involvement of miR827, phosphate and sugar

**DOI:** 10.1186/s12870-025-06786-2

**Published:** 2025-07-03

**Authors:** Daniela Cordeiro, Jorge Canhoto, Sandra Correia

**Affiliations:** 1 Centre for Functional Ecology, Laboratory Associate TERRA, Department of Life Sciences, University of Coimbra, Calçada Martim de Freitas, Coimbra, 3000-456 Portugal; 2 UCIBIO– Applied Molecular Biosciences Unit, Department of Chemistry, NOVA School of Science and Technology, Universidade NOVA de Lisboa, Caparica, 2829-516 Portugal; 3 InnovPlantProtect CoLab, Estrada de Gil Vaz, Elvas, 7350-478 Portugal

**Keywords:** Cell reprogramming, FISH, MiRNAs’ functional analysis, Phosphate-starvation, Somatic embryogenesis, VbMS

## Abstract

Somatic embryogenesis (SE) is a pivotal model for understanding cellular differentiation and totipotency while also offering practical applications for micropropagation and breeding of many crops. Therefore, there is a great interest in understanding the gene regulatory networks underlying cell reprogramming during this process. Accordingly, SE in *Solanum betaceum* Cav. (tamarillo) is a reliable system for studying embryogenic competence acquisition, expression, and maintenance. Since sugar signalling mediates phosphate starvation responses and the phosphate-starvation-induced miR827 was found upregulated in *S. betaceum calli* with no embryogenic potential, it was hypothesised that the long exposure to high sucrose concentrations during subcultures might be responsible for the loss of embryogenic competence, through phosphate-starvation activated mechanisms, such as miRNA upregulation. Indeed, the results obtained showed a higher miR827 expression and lower phosphate levels with decreased embryogenic potential. These results were further confirmed by fluorescent in situ hybridization and virus-based miRNA silencing techniques. However, *PHOSPHATE TRANSPORTER 5*, a known miR827-target gene involved in phosphate transport, was more expressed in long-term maintained callus (LTC). Although LTC and non-embryogenic callus showed lower phosphate levels when compared to embryogenic callus, changing the phosphate availability on culture media did not result in higher embryogenic competence acquisition or expression. The results obtained so far reinforce the hypothesis that phosphate metabolism is a determinant of embryogenic competence commitment and expression during SE. Overall, this work is a step further in understanding the behaviour of long-term conserved cultures, a common bottleneck in the development of fully optimized SE-based protocols for several woody plant species.

**Supplementary Information:**

The online version contains supplementary material available at 10.1186/s12870-025-06786-2.

## Background

Somatic embryogenesis (SE) is a developmental process whereby somatic plant cells are induced through specific stimuli, usually plant hormones or growth regulators, to reprogram, express totipotency, develop into embryos and later into plantlets [1]. As an efficient biotechnology tool, SE is widely used in micropropagation and in the improvement of numerous economically important crops [2, 3]. In addition, and due to the great interest in this technique, it has been applied to characterise the genetic and physiological events occurring during in vitro plant regeneration [4–7].

*Solanum betaceum* (Solanaceae) is a fruit tree commonly known as tamarillo or tree tomato. Due to the yellow to red fruits, with a distinctive sweet and tart flavour, the economic importance of this species has increased [8–10], as represented by the rate of increase in exports in the United States (30.9%) and in Brazil (126.4%) in the last 5 years (www.tridge.com/intelligences/tamarillo/export accessed on 1st May 2025). Also, recent studies showed the high nutritional value of these fruits, rich in vitamins, minerals and bioactive compounds with health-promoting effects [8, 11, 12]. In plant science and biotechnology research, *S. betaceum* has been pointed out as a suitable model system for fundamental studies, including experimental embryology, cell reprogramming and functional genomic analysis, since SE and further plant regeneration is efficiently achieved in this species [13]. Moreover, in this species, cells with embryogenic capacity (embryogenic callus - EC) and cells without that capacity (non-embryogenic callus - NEC) can be induced and obtained under the same conditions in the same explant, and maintained in culture for several years [14]. The establishment and proliferation of embryogenic lines allow the overcoming of the efficient but even time-consuming processes of in vitro explant establishment and dedifferentiation, enabling the continuous and rapid development of the somatic embryos, along with the study of the SE process. This is particularly important for economically important species and/or elite genotypes from which obtaining starting material is difficult. However, with subsequent long-term subcultures (hereafter termed long-term callus, LTC), the EC loses its embryogenic potential, and further embryo development and plantlet formation are impaired.

In recent years, microRNAs (miRNAs) and their target genes have emerged as key regulators of plant growth, development and stress responses [15, 16]. They have also been found involved in the regulation of cell reprogramming processes occurring in plant regeneration [6, 17]. For instance, several studies have highlighted the role of some miRNAs throughout the SE process, mainly by comparison between EC and NEC and at different stages of somatic embryo development [18]. However, the functional role of most miRNAs in embryogenic competence remains largely unknown. Furthermore, only a few studies exist on the miRNA regulation of the embryogenic potential maintenance through subcultures [19–21].

In previously obtained high-throughput sequencing data from small RNAs (sRNAs) libraries, including miRNAs, differential miRNAs expression profiles were revealed among *S. betaceum* SE-induced cell lines with distinct embryogenic abilities (unpublished data). Some miRNAs, such as miR827, were found more expressed in LTC and/or NEC, suggesting their putative involvement in the loss of embryogenic capacity throughout subcultures. As the loss of the embryogenic commitment impairs the efficient use of SE, not only in this species but in others [19–21], miR827 was selected to be functionally characterized and to check its biological relevance in the loss of embryogenic competence. miR827 and its target genes have been reported as playing important regulatory roles in cellular phosphate homeostasis in plants [22, 23]. In phosphate-starvation conditions, miR827 is upregulated and targets *PHOSPHATE TRANSPORTER 5* (*PHT5*), which increases the phosphate accumulation in the cell cytoplasm [24].

To our knowledge, the effect of this miRNA-target gene module on plant embryogenic competence has not yet been investigated. Nevertheless, the importance of phosphate metabolism in cell fate determination has been highlighted in other physiological mechanisms. Along with other elements, phosphate was considered a trigger in somatic cell fate change and hence at the start of the embryogenic process [25]. Indeed, higher phosphate levels have been associated with higher embryogenic and morphogenic responses. For instance, the increase of the phosphate level in the culture media promoted EC induction and proliferation in *Sorghum bicolor* [26], somatic embryo induction, maturation and conversion in *Cymbopogon schoenanthus* [27] and shoot organogenesis in *Hippophae rhamnoides* [28]. In turn, the decrease of the phosphate levels inhibited shoot meristem formation in tobacco [29], as well as phosphate-limiting conditions were found to increase auxin sensitivity and accumulation in *Arabidopsis* [30, 31]. Furthermore, several studies have pointed out an interaction between phosphate and sugar metabolism [32], namely that phosphate-starvation responses are mediated by sugar [33, 34].

Thus, since *S. betaceum* EC is induced and subcultured in media containing high sucrose levels (0.26 M), the research here conducted aimed to address whether sucrose accumulation was involved in the loss of embryogenic competence through phosphate-starvation mediated mechanisms, such as miR827 upregulation. A combination of molecular techniques, including miRNAs and gene expression quantification, in situ hybridization and virus-based microRNA silencing, with physiological assays, such as phosphate and sucrose quantification and evaluation of their effect in culture media, were applied. Overall, miR827 functional characterization will provide new insights into the molecular mechanisms underlying embryogenic competence in this species. Such findings could contribute to the development of more efficient and effective protocols for the micropropagation of economically important plant species.

## Methods

### Plant material and sampling

Three proliferating *S. betaceum* cell lines with distinct embryogenic competencies were used in this assay, namely two-year-old embryogenic callus (EC), ten-year-old non-embryogenic callus (NEC) and eight-year-old long-term callus (LTC, obtained at the same time as NEC); as described in Cordeiro et al., 2024 [35]. Each cell line was obtained from an individual section of a leaf (2–3 weeks) divided into 4, from in vitro proliferating shoots, following the methodology previously described in Correia and Canhoto (2018) [13], and had been maintained in Murashige and Skoog medium [36] (Duchefa Biochemie, Haarlem, The Netherlands), supplemented with 20 µM picloram (Sigma-Aldrich^®^, Missouri, USA) plus 0.26 M sucrose (Duchefa Biochemie), and gelified with 0.25% (w/v) Phytagel™ (Sigma-Aldrich^®^) at pH 5.7. Cultures were maintained at 24 ± 1 °C under dark conditions, by monthly subculture. The embryogenic potential of each cell line was continuously assessed by the ability to develop somatic embryos (i.e., the number of somatic embryos formed).

In silico **analysis**.

### *In silico* analysis

#### *S. betaceum* MiRNA sequence characterization

A selected miRNA sequence, previously found to accumulate in *S. betaceum* LTC and NEC, 5’-UUAGAUGAACAUCAACAAACA-3’, was used to search for similarity in the miRBase database (https://www.mirbase.org/) [37, 38]. The alignment was performed with the Viridiplantae mature miRNA sequences using the BLASTN method [39]. Sequences with a score > 100 and an expectation value of 0.002 were selected. Then, this sequence was characterized according to the online Plant miRNA Encyclopedia (PmiREN: https://www.pmiren.com) [40].

#### miR827-targets prediction

The miR827-target prediction was first performed by literature review. Then, a plant small RNA target analysis server (psRNATarget; https://www.zhaolab.org/psRNATarget/) [41] was used to predict target genes of the mature sequence of miR827 from *S. lycopersicum* obtained from PmiREN (5’-UUUGUUGAUGGUCAUCUAUUC-3’). Default parameters for Schema V2 (2017 release) were used in the target library “*Solanum lycopersicum* (tomato), cDNA, Solanaceae Genomics Network, ITAG 4.0 released on September 06, 2019”.

#### Primers design

The miRNA expression was quantified using the two-tailed reverse transcription quantitative PCR (RT-qPCR) method. Specific two-tailed RT, forward and reverse primers were designed using the mature and star sequences of miR827 from *S. lycopersicum* (PmiREN), following the methodology described in Androvic et al. (2017) [42]. Primers for the mature sequence of miR166a from *S. lycopersicum* (PmiREN019036 and MIMAT0007915 in miRBase) were also designed, to be used as the reference for miRNA quantification in *calli* samples according to [43]. The secondary structure of the two-tailed RT primers was predicted using the RNAfold WebServer (http://rna.tbi.univie.ac.at//cgi-bin/RNAWebSuite/RNAfold.cgi, accessed on 4th July 2022) [44]. Two-tailed RT primers, and forward and reverse primer pairs were checked for dimer formation using the Multiple Primer Analyzer from TermoFisher and Oligo Analysis Tool from Eurofins, respectively.

For miR827-target gene quantification, primers were designed in the most conserved regions of the *S. lycopersicum* sequences available on NCBI, when aligned with other Solanaceae species. Primer design was performed using the primer designing tool from NCBI (https://www.ncbi.nlm.nih.gov/tools/primer-blast/) [45]. *small nucleolar RNA 14* (*snoR14*) and *UBIQUITIN 10* (*UBQ10*) were used as reference genes according to [43].

All oligonucleotides were synthesized by STAB VIDA (Lisbon, Portugal). The specificity of each pair of primers was confirmed by a single band in 1.5% (w/v) agarose gel electrophoresis and by analysis of the melting curves on qPCRs. The sequences of all primers mentioned above and respective melting temperatures are provided in Table S1 (Additional file 1).

### Gene expression analysis

#### RNA extraction

Before RNA extraction, all materials were autoclaved at 121 ºC for 20 min twice, to avoid RNases. ~100 mg frozen callus samples were ground to a fine powder in liquid nitrogen using a mortar and pestle. Total RNA extraction was first performed using the Direct-zol™ RNA MicroPrep (Zymo Research, California, USA), according to the manufacturer’s instructions. Genomic DNA was removed using RNase-free DNase I (Zymo Research). The concentration and quality of total RNA were assessed using a NanoDrop™ One Spectrophotometer (Thermo Scientific™, Thermo Fisher Scientific Inc., Massachusetts, USA). Only RNA samples with A260/A280 ratio between 1.8 and 2.1 and A260/A230 ratio higher than 1.8 were further used for cDNA synthesis.

#### cDNA synthesis

Both miRNA and target gene quantification were performed on the same RNA samples, consisting of three biological replicates from each EC, LTC and NEC. For miR827 quantification, the two-tailed RT reactions were performed with the qScript Flex cDNA Kit (Quantabio, Massachusetts, USA) in multiplex, whenever possible. In a total reaction volume of 10 µl, 500 ng of total RNA and 0.5 µM of each RT primer were used. RT reactions were incubated at 25 ºC for 45 min followed by 5 min at 85 ºC in an Arktik™ Thermal Cycler (Thermo Scientific™). For *PHT5* quantification, RT reactions were performed with the NZY First-Strand cDNA Synthesis Flexible Pack (NZYTech). In a total reaction volume of 10 µl, 500 ng of total RNA and 2.5 µM of oligo(dT) were used. RT reactions were incubated following the manufacturer’s instructions in an Arktik™ Thermal Cycler (Thermo Scientific™).

#### Quantitative PCRs

qPCR reactions were performed with the PerfeCTa SYBR^®^ Green SuperMix (Quantabio), in a total reaction volume of 10 µl containing 0.5 µM of each specific primer. For miR827 qPCRs, 4 µl of 25-fold diluted cDNA template were used and reactions were incubated at 95 °C for 2 min, followed by 45 cycles of 10 s at 95 °C and 30 s at 55 °C. For *PHT5* qPCRs, 4 µl of 10-fold diluted cDNA template were used and reactions were incubated at 95 °C for 3 min, followed by 45 cycles of 15 s at 95 °C, 45 s at 55 °C and 1 min at 70 ºC. The relative expression was calculated according to the Pffafl method [46]. miR166a was used as the reference gene for miR827 qPCRs (annealing at 52 ºC), while *PHT5* qPCRs normalization was performed with *UBQ10* and snoR14 according to Cordeiro et al. [43]. Relative expression was compared and statistically analysed by t-test or one-way ANOVA (*p* < 0.05), followed by Tukey’s multiple comparisons tests. A Pearson correlation coefficient was computed to assess the linear relationship between the relative expression of the miRNA and the putative target gene (*p* < 0.05).

In situ **localization of miR827**.

In situ localization of miR827 was conducted following the methodology described by Huang et al. (2019) [47], with some modifications, and using conventional oligonucleotide probes as described by Hernández-Castellano et al. (2017) [48]. The reverse complement sequence of the miR827 sequence differently expressed in *S. betaceum* was used to design the probe (Table S2, Additional file 1). As mature miR166a was found to be the best reference for qPCR data normalization in *calli* samples [43], a probe with the reverse complement of this miRNA was used as a positive control and a scrambled probe with the sequence of this miRNA in sense was used as a negative control. U6 was also used as a positive control, given that it has been used in other miRNA expression analyses as an internal reference gene [49]. Conventional oligonucleotide probes were synthesized with digoxigenin (DIG) labelling at 3’ end by Eurofins Genomics (Ebersberg, Germany). All buffers and solutions were previously prepared with diethylpyrocarbonate (DEPC)-treated water and all material was cleaned with RNase Cleaner (NZYTech) before autoclaving, to avoid any RNase activity.

Samples of EC, LTC and NEC were collected, immersed in 4% paraformaldehyde in PHEM buffer (5 mM HEPES, 60 mM PIPES, 10 mM EGTA and 2 mM MgCl_2_, at pH 7) and subjected to vacuum infiltration for 15 min, before overnight incubation, under continuous gentle agitation, at 4 °C. After fixation, samples were dehydrated by incubation in increasing sucrose concentrations (10, 20 and 34% (w/v) prepared in phosphate-buffered saline (PBS). Incubations in sucrose were performed overnight, under continuous gentle agitation, at 4 °C. Dehydrated samples were placed in molds with Tissue-Tek^®^ O.C.T. Compound (Sakura Finetek USA, Inc., California, USA) and frozen in isopropanol bath cooled in liquid nitrogen. The molds were stored at -80 °C until further processing. The Tissue-Tek^®^ blocks were sectioned with 10 μm thickness at 20 °C using a cryostat (Leica CM 3050 S, Leica, Wetzlar, Germany). Tissue sections were placed on Fisherbrand™ Tissue Path SuperFrost™ Plus Gold Slides (Thermo Fisher Scientific), which were stored at -80 °C until further processing.

The tissue sections were marked using a ReadyProbes™ Hydrophobic Barrier PAP Pen (Thermo Fisher Scientific), before protease (50 mg/mL in TE buffer; Sigma-Aldrich^®^) digestion, by incubation at 37 °C for 10 min in a moist chamber. Samples were then treated with 0.2% glycine (Sigma-Aldrich^®^) for 10 min followed by two washes in PBS buffer for 5 min. A TEA treatment (Triethanolamine, Sigma-Aldrich^®^, HCl and acetic anhydride) was performed for 1 h with gentle shaking. After two washes in PBS buffer for 5 min and one with water for 1 min, samples were dehydrated by going through an ethanol series of 10%, 30%, 50%, 70%, 80% and 95% (vol/vol) for 30 s each and 100% ethanol for 1 min twice. Samples were allowed to dry completely and incubated with hybridization buffer (as described in [47]) in a wet chamber, containing saline-sodium citrate buffer (SSC, Merck) 4 x and 50% formamide, for 1 h. 100 pmol of DIG-labelled probe in 10 µl were mixed in 10 µl formamide (Merk) and incubated at 85 ºC for 5 min. This mix was then placed on ice immediately before use and further added to 80 µl of hybridization buffer. 20 µl of hybridization mix was applied to each tissue section on the slides, for hybridization at 54 ºC or 56 ºC overnight in a wet chamber. A hybridization mix without a probe was used as the control. Hybridized slides were washed twice using 0.2 SSC, blocked in blocking buffer (1% blocking reagent in Tris-buffered saline (TBS) and washed with washing buffer (0.5% (w/v) BSA and 0.3% Triton X-100 (v/v) in TBS buffer) for 1 h each. Primary antibody incubation was performed with anti-DIG Fab fragments (from sheep, cat. no. 11214667001, Roche, Sigma-Aldrich^®^) diluted 1:100 in washing buffer at 4 °C overnight. Samples were then washed four times (15 min each) with washing buffer. Secondary antibody (Alexa Fluor™ 680 donkey anti-sheep IgG (H + L) A21102, Thermo Scientific™) diluted 1:200 in washing buffer was incubated at 4 °C overnight, followed by four washes in washing buffer (15 min each wash). After a final wash in the TBS buffer, slides were mounted with the Dako fluorescence mounting medium (Sigma-Aldrich^®^).

Confocal images were acquired with a point scanning confocal microscope Zeiss LSM 710 AxioOberserver (Zeiss, Göttingen, Germany), with a 63x oil objective. Callus autofluorescence was used to detect cell structure, by excitation with a laser of 488 nm wavelength (green). Laser excitation of 633 nm wavelength (red) was used for the secondary antibody immunofluorescence signal. Optical sections were captured with ZEN imaging software. Maximum projection images were analysed using the same settings, for a better and more accurate comparison of signal intensity. The immunofluorescence signal was calculated using the ratio of the integrated density of the red signal (miRNA, arbitrary units (AU) to the green area (callus autofluorescence, µm^2^) with Fiji software [50].

### Virus X-based miR827 Silencing

To evaluate the function of the miR827, the virus-based microRNA silencing (VbMS) approach described by Zhao et al. (2020) [51] was followed. Briefly, short tandem target mimic molecule (STTM) and cloning primers were designed (F: CGACGACAAGACCGTTGTTTGTTGATCTAGTTCATCTAAGTTGTTGTTGTTATGGT and R: GAGGAGAAGAGCCGTTTAGATGAACTAGATCAACAAACAATTCTTCTTCTTTAGACCAT), using the reverse complement of the miR827 sequence differently expressed in *S. betaceum*. STTM-827 fragment was then amplified, purified and polyadenylated. PVX-LIC plasmid was digested with *Sma*I, purified and amplified with T4 DNA polymerase and dTTP. STTM-827 sequence was cloned into the PVX-LIC vector using an LIC reaction. The LIC reaction product was transformed into the *E. coli* strain DH5α (NZYTech) and plated on an LB plate containing 50 µg/ml kanamycin. Positive colonies were verified by PCR, using the forward universal primer for the PVX-LIC vector (5’-GTGTTGGCTTGCAAACTAGAT-3’; Zhao et al. (2020) in combination with the reverse primer for STTM cloning, followed by sequencing. PVX-STTM-827 plasmids were isolated using the NZYMiniprep kit (NZYtech). Plasmids were transformed into *Agrobacterium* strain EHA105. *Agrobacterium* colonies were verified by PCR using the same primers. LTC samples were transformed with *Agrobacterium* containing the PVX-STTM-827 plasmids, following the methodology previously described in Cordeiro et al. (2023a). Following the optimized protocol, transformants were selected in increasing kanamycin concentrations (30, 50 and 100 mg/L). The gDNA of three biological replicates of kanamycin-resistant LTC was extracted using the NucleoSpin™ Plant II kit (Macherey-Nagel™), following the manufacturer’s instructions. Then, the presence of STTM-827 was verified by PCR, using the forward primer for PVX-LIC vector and the reverse primer for STTM-827 cloning with an annealing temperature of 56 ºC. Isolated plasmids were used as the positive control, DNA from a non-transformed callus was used as a negative control and a non-template control was included. To confirm transformation and quantify the miR827 expression, qPCR analysis was carried out on the RNA extracted from at least three biological replicates of kanamycin-resistant LTC. The methodology followed was the one described in the “Gene expression analysis” section, except that non-transformed LTC was used as the control sample.

### Physiological assays

#### Phosphate quantification

Inorganic phosphate (Pi) and total phosphate (P) contents were analysed using the method developed by Ames (1966) [52], following the modifications of Chiou et al. (2006) [53]. Before use, glass tubes were filled with concentrated sulphuric acid overnight, followed by rinses thoroughly. ~100 mg of fresh *calli* tissue was frozen and ground with liquid nitrogen. The fine frozen powder was homogenized with 10 µL/mg tissue of extraction buffer (10 mM Tris, 1 mM EDTA, 100 mM NaCl, 1mM β-mercaptoethanol, and 1mM phenylmethylsulfonyl fluoride, pH 8.0). 100 µL of the homogenized sample was mixed with 900 µL of 1% glacial acetic acid and incubated at 42 °C for 30 min. From this mixture, 100 µL were transferred to a 13 × 100 mm glass tube for total P assay and the other 900 µL were centrifuged at 13,000 g for 5 min. 300 µL of the supernatant was mixed with 700 µL of assay solution (0.35% NH_4_MoO_4_, 0.86 N H_2_SO_4_ and 1.4% ascorbic acid) and incubated at 42 °C for 30 min. 300 µL of water was used as blank. The solution was transferred to a new cuvette and the absorbance was read at 820 nm. Pi content was presented in nmol/mg FW, considering that 10 nmol of Pi results in an A820 of 0.260 [52]. For the total P assay, 30 µL of 10% Mg(NO_3_)_2_ in 95% ethanol was added to the 100 µL aliquot in the glass tube. The tubes were shaken over a strong flame and samples were dried and flamed to ash. After cooling, 300 µL of 0.5 N HCl was added. Tubes were capped with parafilm and incubated at 65 °C for 30 min. After cooling, 700 µL of assay solution was added and the described above for Pi was followed. Total P content was presented in nmol/mg FW, considering that 10 nmol of organic phosphate results in an A820 of 0.240 [52]. Three biological replicates of EC, LTC and NEC and three technical replicates from each were analysed. Pi and total P were also quantified in kanamycin-resistant callus resulting from the miR827 VbMS assay. Data were statistically analysed by t-test or one-way ANOVA (*p* < 0.05), followed by Tukey’s multiple comparisons tests.

#### Effect of Pi concentration on SE induction

To test the effect of the Pi concentration on the induction of the SE process in *S. betaceum* (described in “Plant material and sampling” section), different Pi concentrations were used on the induction medium. Besides the Pi concentration described for MS medium (1.25 mM), which was used as the control, 0, 0.6, 2.5 and 5.0 mM Pi were also tested. After twelve weeks of incubation, the percentage of explants forming EC and NEC or with no response (N) was recorded from at least twelve biological replicates tested per condition.

#### Effect of Pi concentration on somatic embryo development

To test the effect of the Pi concentration on the somatic embryo development in *S. betaceum*, clusters of 100 mg proliferating EC were incubated in the development medium supplemented with KH_2_PO_4_ to reach a Pi concentration of 12.5 mM (tenfold that the described for MS medium). EC incubated in the development medium (1.25 mM Pi) was used as the control. After five weeks, the whole tissue, from at least nine biological replicates, was weighed and transferred to the conversion medium. After another five weeks, tissue with developing and formed somatic embryos was subcultured in a fresh medium. After four weeks, the number of somatic embryos developed per the initial 100 mg EC was recorded in six biological replicates. Somatic embryos were considered those with an embryo axis containing SAM and RAM. Data were statistically analysed by t-test or one-way ANOVA (*p* < 0.05), followed by Tukey’s multiple comparisons test.

#### Sucrose quantification

Soluble sugars were extracted following the protocol described by Leach and Braun (2016) [54]. 100 mg of fresh *calli* tissue (EC, LTC and NEC) were frozen and ground with liquid nitrogen. The fine frozen powder was homogenized with 1 mL of methanol: chloroform: water (12:5:3) and incubated at 50 °C for 30 min. Samples were centrifuged for 5 min at 14,000 rpm. The supernatant was recovered and stored on ice, while the pellet was re-extracted using 1 ml of MCW two more times. The volume of pooled supernatant was recorded and 0.6 volumes of water were added. Samples were vortexed and centrifuged at 4.650 x g for 5 min. The volume of the aqueous phase was recorded and stored at -20 °C for later quantification. Sucrose quantification was performed using the anthrone method described by Handel (1968) [55]. Aliquots of 50 µL from *S. betaceum calli* soluble sugar extraction were used and 50 µL of water was added to each. For the standard curve, 100 µL of 0, 0.2, 0.4, 0.6, 0.8 and 1 mg/mL of sucrose were taken. 100 µL of 30% aqueous KOH was added to all tubes, followed by incubation at 100 °C for 10 min using marble to cap the tubes. After cooling, 3 mL anthrone reagent was added and the tubes were incubated at 40 °C for 12 min. The absorbance was read at 620 nm. Sucrose content was presented in µmol/g FW, considering the calibration curve, the dilution of the sample and the volume of the aqueous phase. At least five biological replicates of EC, LTC and NEC and three technical replicates from each were analysed. Data were statistically analysed by one-way ANOVA (*p* < 0.05), followed by Tukey’s multiple comparisons tests.

#### Effect of sucrose on embryogenic competence

The effect of sucrose on the embryogenic competence expression was tested by three approaches.

First, clusters of 100 mg proliferating EC were incubated in reduced sucrose levels (3%, maintaining the auxin) or in an auxin-free medium maintaining the 0.26 M sucrose. EC incubated in the proliferation medium (with auxin and 0.26 M sucrose) was used as a control. Proliferation and presence or absence of embryo development were observed in seven biological replicates of each treatment/condition after six weeks.

Secondly, clusters of 100 mg proliferating EC were incubated in the development medium maintaining the sucrose levels (0.26 M). After six weeks, EC was incubated in the conversion medium (0.087 M sucrose) or subculture in a fresh medium (maintaining the 0.26 M sucrose) and transferred to light conditions. EC incubated in the development medium (0.117 M sucrose), followed by incubation in the conversion medium (0.087 M sucrose) was used as a control. The number of developed somatic embryos was recorded from at least four biological replicates of each condition.

Thirdly, proliferating EC was subcultured with gradually reduced sucrose levels, under the same proliferation conditions. First, clusters of 100 mg proliferating EC were incubated in 0.204 M sucrose for three weeks, after which fresh weight was recorded. Then, EC was incubated in 0.146 M sucrose for another three weeks, followed by incubation in an auxin-free medium containing 0.087 M sucrose and transferred to light conditions after four weeks. EC proliferating in the proliferation medium was used as the control. Six biological replicates were used per treatment/condition. Data were statistically analysed by t-test (*p* < 0.05).

## Results

### Sbe-miR827 characterization

**Fig. 1:**
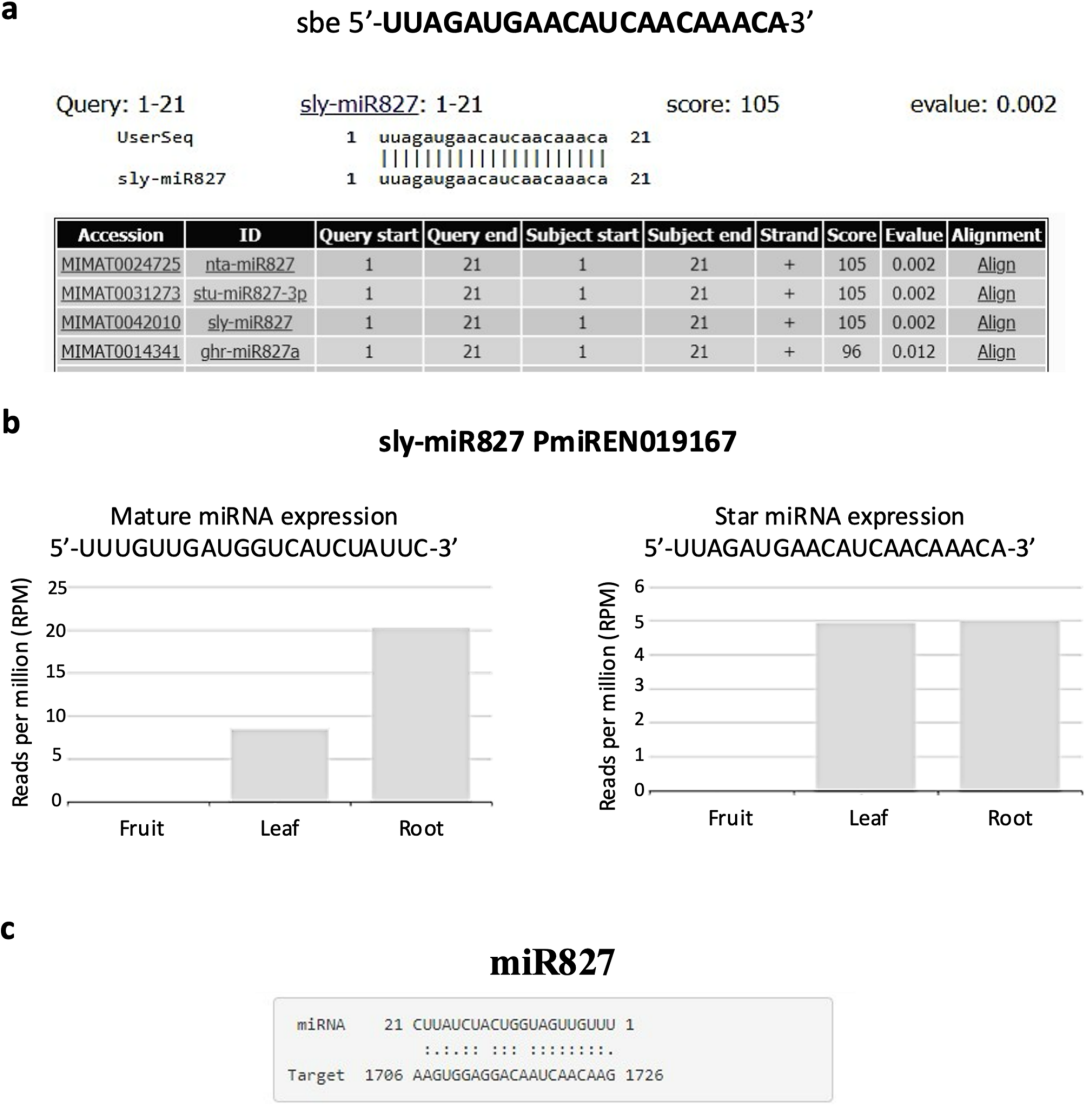
*S. betaceum* miR827 in silico characterization. **a** Alignment of *S. betaceum* sequence with Viridiplantae in miRBase. **b** Expression levels of miR827 from *S. lycopersicum* in PmiREN. **c** Target prediction of the mature sequence of *S. lycopersicum* miR827 using psRNATarget

According to miRBase, the 5’-UUAGAUGAACAUCAACAAACA-3’ sequence found in *S. betaceum* aligned with mature sequences miR827 from *S. lycopersicum* (accession MIMAT0042010, Fig. 1a), miR827-3p from *S. tuberosum* (accession MIMAT0031273) and miR827 from *N. tabacum* (accession MIMAT0024725). In PmiREN, the miR827 family has 69 miRNA gene entries, of which one is from *S. lycopersicum*. In Solanaceae, miR827 is annotated in *Capsicum annuum*, *S. habrochaites*, *S. lycopersicum*, *S. melongena*, *S. pennellii*, *S. pimpinellifolium*, *S. tuberosum* and *N. tabacum*. In *S. lycopersicum*, miR827 corresponds to the locus accession PmiREN019167. The star sequence of this miRNA in PmiREN corresponds to the one found differentially expressed in *S. betaceum* (the mature sequence in miRBase, 5’-UUAGAUGAACAUCAACAAACA-3’). This sequence is identical to the miR827 star sequence from *S. tuberosum* and *S. melongena*, to the miR827 mature sequence from *S. pennellii* and the mature sequences of miR827a and b from *N. tabacum* in PmiREN (locus accession PmiREN019904, PmiREN019434, PmiREN019577, PmiREN012858 and PmiREN012859, respectively). In turn, the miR827 mature sequence from *S. lycopersicum* (5’-TTTGTTGATGGTCATCTATTC-3’), in PmiREN, is identical to the miR827 mature sequence from *S. tuberosum* and *S. melongena*, to the miR827 star sequence from *S. pennellii* and the star sequences of miR827a and b from *N. tabacum*. According to PmiREN, the miR827 mature sequence from *S. lycopersicum* is more expressed, in reads per million, than the star one. Both sequences are more expressed in the root than in the leaf (Fig. 1b, Table 1).

In silico miRNA target prediction was performed to predict potential target genes of miR827, using the online resource for plant sRNA sequences, the psRNATarget. The mature sequence of *S. lycopersicum* miR827 was expected to target 160 genes. From these, Solyc08g080200 was found to be putatively targeted through inhibition of the translation by complementarity in one region, with an expectation value of 4.0 (Fig. 1c). By doing a BLAST in NCBI, this sequence was 100% identical to the sequence ID XM_004245610.4, which is predicted to be an SPX domain-containing membrane protein At4g22990-like (LOC101248506), corresponding to *PHOSPHATE TRANSPORTER 5* (*PHT5*).

**Table 1:** Effect of sucrose concentration on the expression of the embryogenic competence

EMBRYO DEVELOPMENT^1^ Dark conditions	EMBRYO CONVERSION^1^ Light conditions	Number of somatic embryos^2^	Observations
0.26 M sucrose	0.26 M sucrose	57.8 ± 10.2	No conversion
0.26 M sucrose	0.087 M sucrose	31.5 ± 6.0	Higher proliferation
0.117 M sucrose	0.087 M sucrose	11.9 ± 11.4	Emblings developed

^**1**^Auxin-free MS medium; ^**2**^Values are the mean ± SD number of somatic embryos developed per 100 mg of EC, of at least four biological replicates

### The decrease of the embryogenic potential is followed by an upregulation of sbe-miR827

**Fig. 2:**
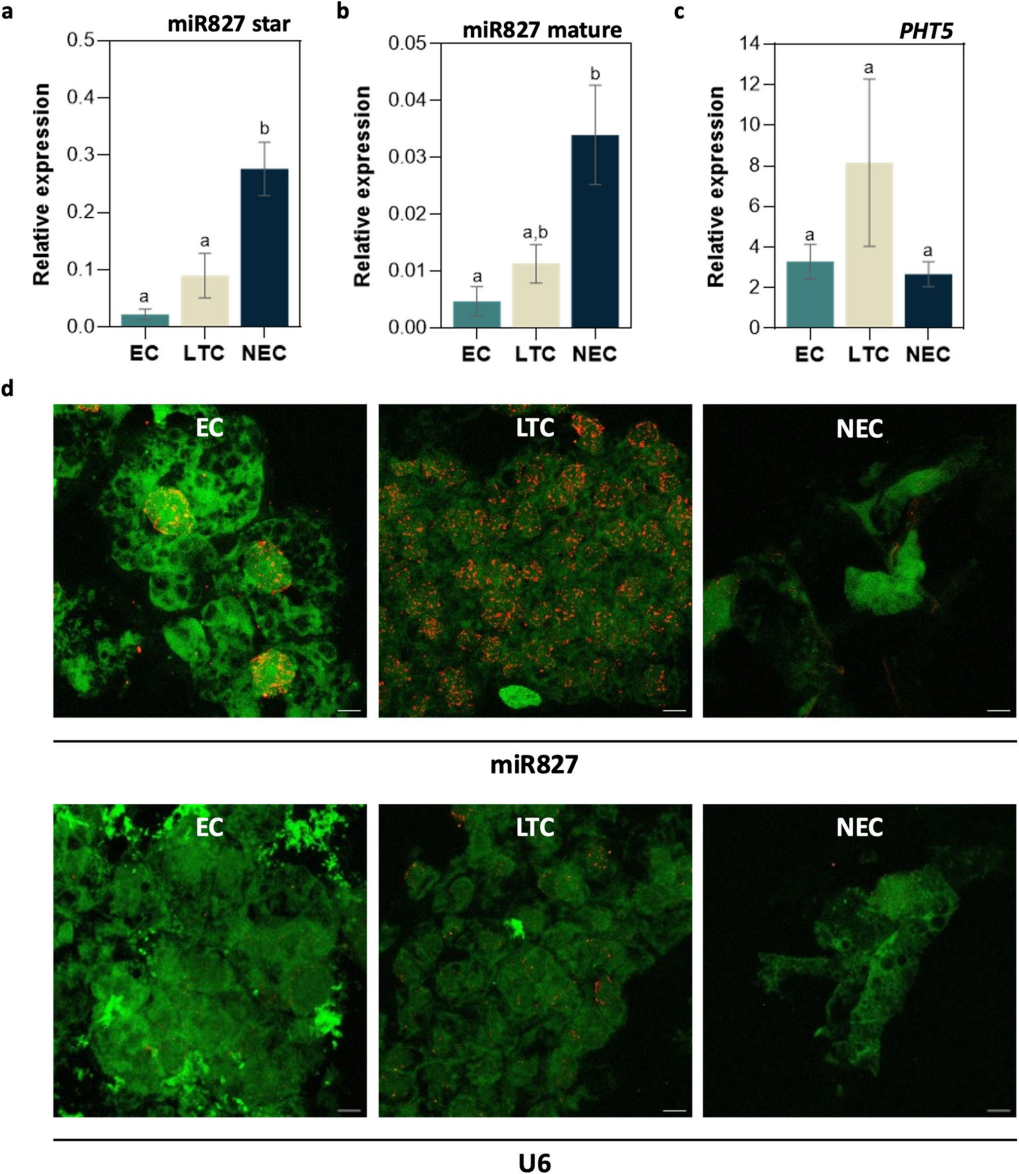
Expression profiles of the star (**a**) and mature (**b**) sequences of miR827, according to PmiREN, in *S. betaceum calli* determined by two-tailed RT-qPCR. EC– embryogenic callus, LTC– long-term callus, NEC– non-EC. miRNAs relative expression was calculated according to the Pffafl method, in which miR166 was used as the reference gene. Each column represents the mean ± SEM of three biological replicates. **c** Expression profile of the miR827-putative target gene (*PHT5*) in *S. betaceum*-induced cell lines. EC– embryogenic callus, LTC– long-term callus, NEC– non-EC. Relative expression was calculated according to the Pffafl method, in which *UBQ10* and *snoR14* were used as the reference genes. Each column represents the mean ± SEM of three biological replicates. Different letters indicate significant differences between treatments by one-way ANOVA (*p* < 0.05) followed by Tukey’s test (**a**, **b** and **c**). **d** Representative confocal microscopy images of fluorescent in situ localization of miR827 and U6 (red signal) over the autofluorescence of *S. betaceum calli* samples (in green). Bars represent 10 μm

Using the two-tailed RT-qPCR method, the expression of star and mature sequences of miR827 from *S. lycopersicum* were quantified in *S. betaceum* EC, LTC and NEC samples. In general, the miRNA showed low relative expression levels. Star and mature strands revealed similar expression patterns. Nevertheless, the star sequence (Fig. 2a) was more expressed than the mature one (Fig. 2b), which is in accordance with the sequencing data. miR827 revealed a higher expression with the decrease of the embryogenic potential, with NEC showing the highest relative expression. For both strands, miR827 was statistically significantly more expressed in NEC than in EC. miR827 star was also more expressed in NEC than in LTC (Fig. 2a).

To confirm the biological relevance of the target prediction, the putative miR827-target gene was quantified in *S. betaceum* EC, LTC and NEC samples. Primers were designed in the sequences found above (Solyc08g080200, *PHT5*), in the most conserved regions among other Solanaceae species. *PHT5* showed higher relative expression in LTC, although no statistically significant differences were found among *calli* samples. *PHT5* showed an inverse expression pattern compared to the miR827, being more expressed in LTC than NEC (Fig. 2c). Nevertheless, no significant correlation was found between miR827 and *PHT5* relative expression levels.

In miR827 localization by fluorescent in situ hybridization (FISH), no signal was detected in samples hybridized with antisense miR166a probe, whereas similar levels of U6 immunofluorescence signal were detected among *calli* samples (Fig. 2d). Fluorescent in situ hybridization of miR827 was accomplished, by the detection of a red signal (miRNA probe) over the green autofluorescence of *S. betaceum calli* (Fig. 2d). miR827 showed a significantly higher signal with wider distribution in LTC samples than in EC (Fig. 2d), in accordance with qPCR results. However, contrary to that data, the miR827 immunofluorescence signal was also more intense in LTC compared to NEC. No signal was observed when the probe, antibody primary or secondary was omitted (data not presented).

### Lower embryogenic competence is related to P-starvation

**Fig. 3:**
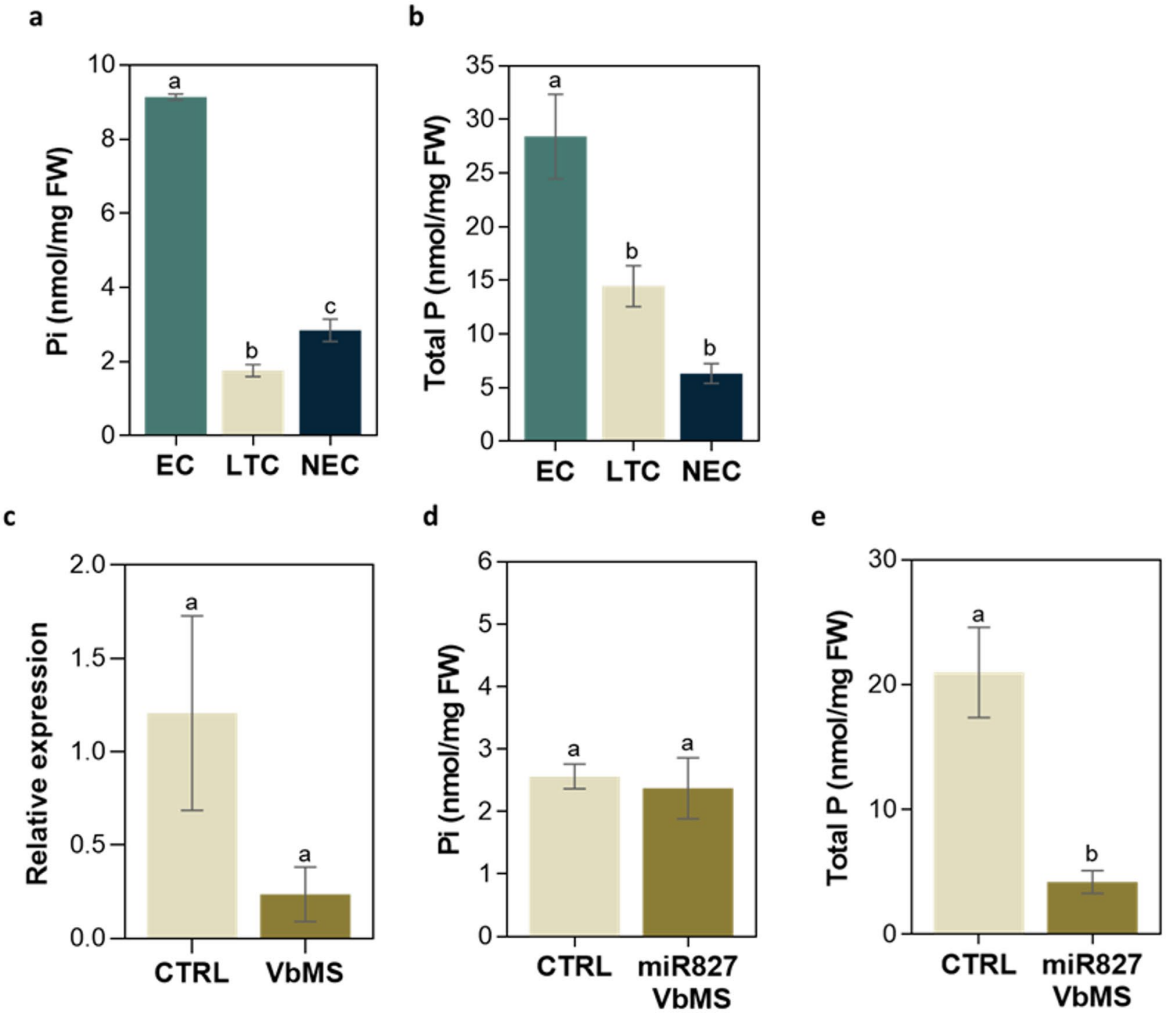
Evaluation of Pi and total P in the different *S. betaceum calli*, and in LTC after miR827 VbMS. Quantification of Pi (**a**) and total P (**b**) concentration in embryogenic callus (EC), callus that has lost its embryogenic capacity (LTC) and non-EC (NEC). **c** miR827 relative expression quantification in LTC (CTRL) and LTC from VbMS with PVX-STTM plasmids. Relative expression was calculated according to the Pffafl method, in which miR166 was used as the reference gene and non-transformed LTC as the control sample. Pi (**d**) and total P (**e**) quantification in long-term callus (LTC; CRTL) and kanamycin-resistant LTC from miR827 VbMS assay. Each column represents the mean ± SEM of at least three biological replicates. Different letters indicate significant differences between treatments by one-way ANOVA and Tukey’s test at *P* ≤ 0.05 (**a** and **b**) and by t-test at *p* < 0.05 (**c**, **d** and **e**)

Pi concentration (nmol/mg fresh weight) quantified in EC (9.1 ± 0.3) was fivefold that of LTC (1.8 ± 0.7) and threefold that of NEC (2.8 ± 1.3; Fig. 3a). In turn, the total P content was found lower the lower embryogenic competence (Fig. 3b).

To further functional characterize miR827, a virus-based microRNA silencing (VbMS) approach was performed. miRNA silencing was conducted in LTC, in which this miRNA was found upregulated compared to EC, following the methodology previously described in Cordeiro et al. (2023) [56]. qPCR analysis was then conducted in kanamycin-resistant callus, to confirm STTM-827 insertion and to quantify miR827 expression. An effective downregulation of miR827 was verified, besides with no statistically significant differences (Fig. 3c); even though in some tested samples, no miR827 relative expression was detected, indicating higher or almost complete miR827 silencing.

By downregulation of miR827, no significant differences were found in the Pi levels measured, while lower total P concentration was found compared to the control (Fig. 3d and e, respectively).

### Effect of Pi concentration on SE induction and embryo development

**Fig. 4:**
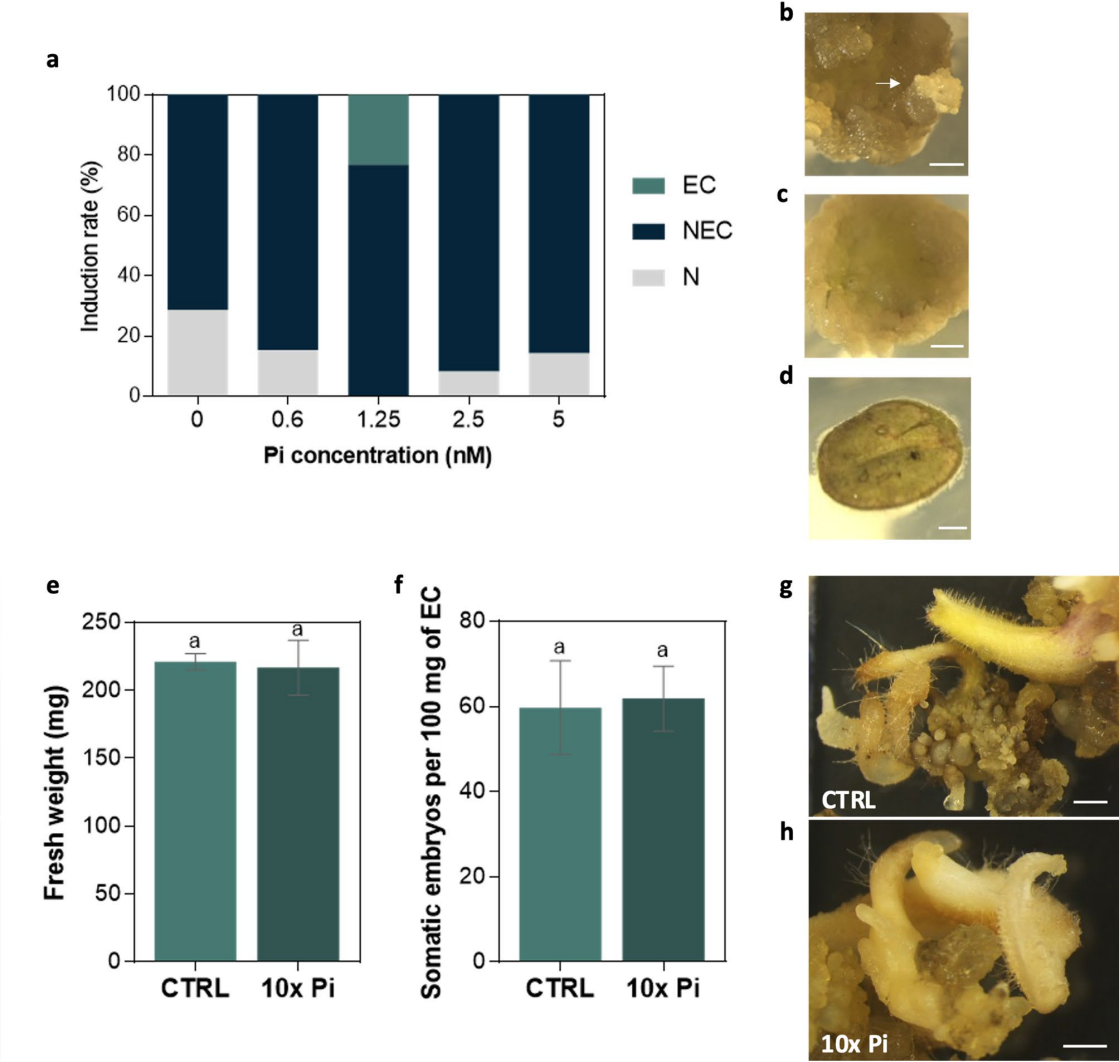
Effect of Pi concentration on SE induction and embryo development. **a** Induction responses of leaf explants to different Pi concentrations in the SE induction medium from at least twelve biological replicates. **b** Embryogenic callus (EC) formation (arrow). **c** Non-EC (NEC). **d** Non-responsive explant (N). Bars represent 1 mm. **e-h** Effect of Pi concentration on somatic embryo development. **e** Fresh weight of EC incubated in development medium (CTRL) and Pi-enriched medium (10x Pi, 12.5 mM) for five weeks. **f** Number of somatic embryos per 100 mg of EC incubated in development medium (CTRL) and Pi-enriched medium (10x Pi, 12.5 mM). **e** and **f** Each column represents the mean ± SEM of at least nine and six biological replicates, respectively. No significant differences were found by t-test at *p* < 0.05. **g** Developing somatic embryos in the proliferation medium. **h** Developing somatic embryos in Pi-enriched medium (10x Pi, 12.5 mM). Bars represent 1 mm

Leaf explants dedifferentiated in all media tested with different Pi concentrations, however, most only formed NEC (Fig. 4a). EC induction (Fig. 4b) was only achieved in the medium with 1.25 mM, the concentration described for the MS medium. These results showed that increasing the Pi concentration does not result in a higher EC induction rate, at the concentrations tested.

Also, the effect of Pi concentration on *S. betaceum* somatic embryo development was tested. A development medium enriched in Pi (12.5 mM, tenfold that described in the MS medium) was used. However, EC incubation at a tenfold Pi concentration resulted in a similar proliferation rate and an equal number of somatic embryos formed compared to the control (Fig. 4e-h).

### Effect of sucrose concentration on embryogenic competence

**Fig. 5:**
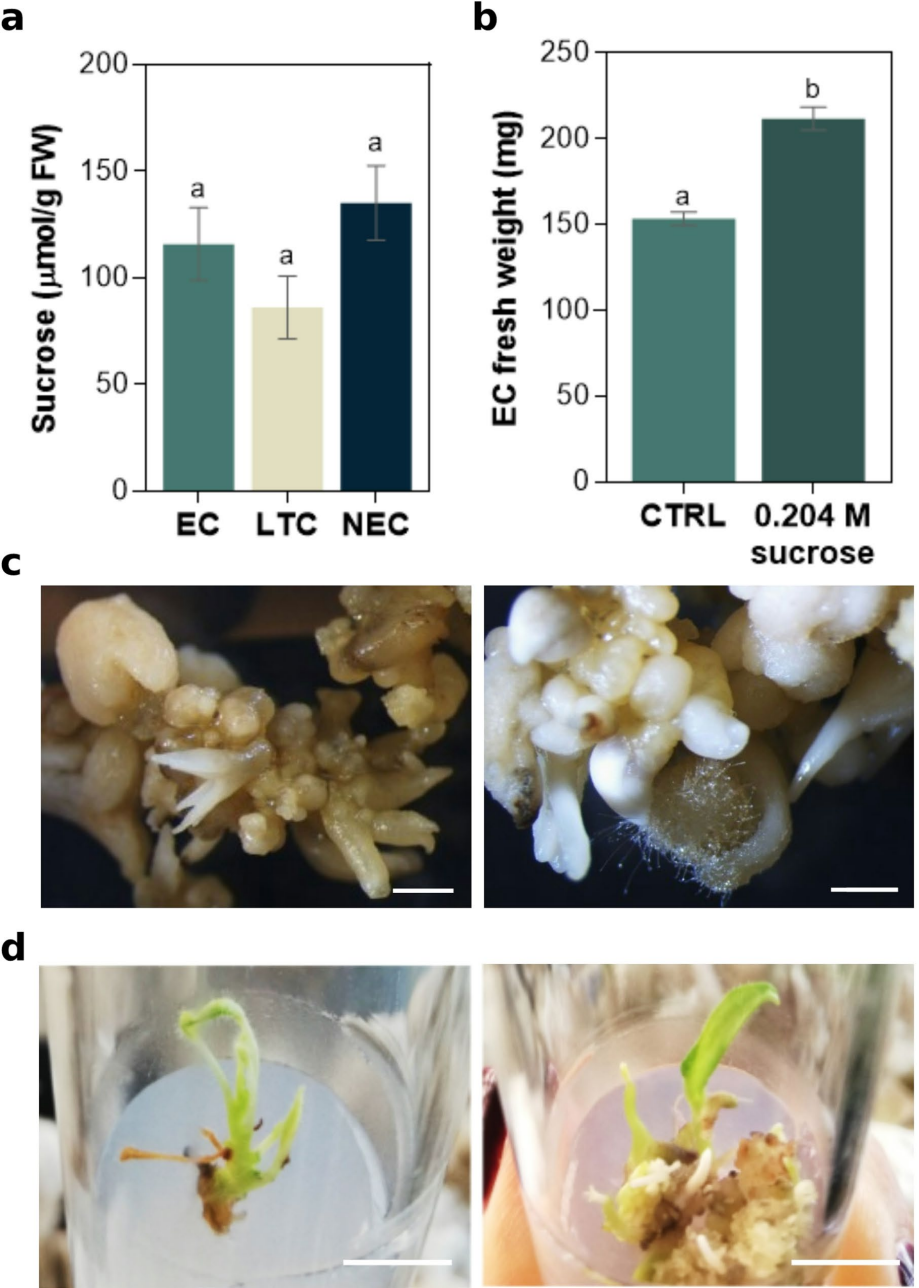
Effect of sucrose concentration on embryogenic competence. **a** Sucrose concentration in *S. betaceum* embryogenic callus (EC), callus that lost its embryogenic capacity (LTC) and non-EC (NEC). Each column represents the mean ± SEM of at least five biological replicates. **b** EC fresh weight after three weeks of incubation in proliferation medium with 0.26 M (w/v) sucrose (CTRL) and 0.204 M sucrose. Each column represents the mean ± SEM of six biological replicates. Different letters indicate significant differences between treatments by one-way ANOVA (*p* < 0.05) followed by Tukey’s test (**a**) and by t-test at *p* < 0.05 (**b**). **c** Developing somatic embryos in auxin-free medium with high sucrose levels (0.26 M). Bars represent 1 mm. **d** Somatic embryo conversion from EC incubated in auxin-free medium with 0.117 M sucrose and then reduced to 3%. Bars represent 1 cm

*S. betaceum calli* are usually induced and proliferated in high sucrose levels (0.26 M). To find whether the long exposure to this high exogenous sucrose concentration is resulting in accumulation by the cells, the sucrose content was quantified in EC, LTC and NEC. However, regardless of callus age (time in culture) and its embryogenic capacity, the results showed similar sucrose concentrations among the three cell lines (Fig. 5a). Thus, the high sucrose levels that *calli* were exposed to in the subsequent subcultures did not translate into higher sucrose contents inside the cells.

Although EC, LTC and NEC showed similar sucrose concentrations, the effect of the sucrose content of the culture medium on the expression of the embryogenic competence was tested, in the presence and absence of auxin.

By reducing the sucrose levels of the proliferation medium from 0.26 to 0.204 M, a significantly higher proliferation rate was achieved, resulting in 58.3 ± 7.8 mg more fresh weight (Fig. 5b). However, no somatic embryo development was observed after the sucrose weaning assay.

*S. betaceum* EC is induced and proliferated in the presence of auxin (20 µM Picloram) and high sucrose levels (0.26 M). By auxin removal and sucrose maintenance, a higher proliferation rate was observed, compared to the control. Moreover, somatic embryo development was achieved in this medium (Fig. 5c). In contrast, EC browning was observed when auxin was kept and sucrose reduced to 0.087 M. This confirms that auxin blocks the expression of embryogenic competence.

EC was incubated in the development medium maintaining the sucrose levels (0.26 M), followed by transfer to light conditions and maintaining the 0.26 M sucrose or reducing it to 0.087 M. Somatic embryo development was achieved in all conditions tested (Table 1). Even though, a higher number of somatic embryos occurred when 0.26 M sucrose was maintained for embryo development, in an auxin-free medium (Fig. 5c). The highest number of somatic embryos was reached by maintaining the high sucrose levels in both incubation media (Table 1). However, somatic embryo conversion (emblings development) was only observed in the control treatment (Fig. 5d). In the absence of auxin and compared with the control, a higher proliferation rate was observed when EC was incubated in 0.26 M and then in 0.087 M sucrose.

## Discussion

The primary objective of this work was to identify expression trends and potential molecular players associated with the gradual loss of embryogenic potential. Accordingly, functional characterization of miR827 on phosphate starvation mediated mechanisms, and their potential involvement in the embryogenic capacity maintenance in *S. betaceum*, was assessed using a combination of molecular techniques, including miRNAs and gene expression quantification, in situ hybridization and virus-based microRNA silencing, with physiological assays, such as phosphate and sucrose quantification and evaluation of their effect in culture media. Several of those methods were applied for the first time in *S. betaceum*, which is a non-model species, with limited functional genomics tools available. The challenges in such approaches and the results obtained are discussed in the following sections.

miR827 family is angiosperm-specific, being described in dicotyledons and monocots [24]. miR827 is considered Pi-starvation responsive, since it is specifically induced in response to low Pi levels in several species of the Poaceae (*Brachypodium distachyon*, *Hordeum vulgare*, *Oryza sativa* and *Zea mays*) [22, 24], Brassicaceae (*Arabidopsis thaliana* and *Brassica rapa*) [24], Cucurbitaceae (*Citrullus lanatus*) [24] and Solanaceae (*Solanum lycopersicum* and *Nicotiana benthamiana*) [24] families.

Phosphorus is a macronutrient essential for plant growth and development. Being part of fundamental macromolecules and nucleotides, it is involved in many biochemical and physiological processes, including metabolic pathways and regulation of enzymatic reactions [57]. The regulation of Pi homeostasis in plants is a complex process, that involves Pi uptake, remobilization, storage and recycling mechanisms [58]. The P uptake is carried out in plant roots, in which Pi is absorbed from the soil by Pi transporters, whereas the Pi storage occurs in the vacuoles [59]. These are tightly regulated processes, and plants have developed several strategies to cope with changes in cellular Pi concentrations [60]. For instance, in Pi deficiency, the expression of Pi transporters is regulated by several transcription factors and microRNAs, including the miR827 upregulation [24].

Unlike most plant miRNAs, miR827 does not conservatively target homologous genes in the plant kingdom. Instead, miR827 conservatively targets *PHT5* homologs in most angiosperms, preferentially targets *NITROGEN LIMITATION ADAPTATION* (*NLA*) homologs in Brassicaceae and the related Cleomaceae, and lost its regulatory role in legumes [24]. *PHT5* codes for a protein family containing the SPX (SYG1/PHO81/XPR1) domain, responsible for Pi transport into the vacuole to mediate Pi storage or remobilization [61]. Under Pi deficiency, miR827 upregulation is induced, resulting in the post-transcriptional cleavage of the *PHT5* transcript. Hence, Pi is not transported into the vacuole and accumulates in the cytoplasm [24].

In this work, *S. lycopersicum* was used as a reference genome for in silico miRNA target prediction due to its taxonomic proximity with *S. betaceum*. In that analysis, miR827-*PHT5* targeting was predicted. Thus, for miR827-*PHT5* targeting validation in *S. betaceum* and further functional characterization of the biological role of this miRNA in embryogenic competence, miR827 and *PHT5* qPCR analysis and miR827 in situ hybridization and silencing were employed (further discussed).

The expression of miR827 was quantified in *S. betaceum* EC, LTC and NEC samples by qPCR, the gold standard method for differential expression validation. Although the RNA-seq approach was considered poor precise in the quantification of low abundant miRNAs [62], qPCR results validated the sRNA-seq data previously obtained.

The present results pointed to miR827 as a negative regulator of the totipotency expression in *S. betaceum*, given its upregulation in cells with no embryogenic potential (both LTC and NEC). By contrast, miR827 is specifically expressed in the globular embryo stage in longan and, particularly, the miR827-5p sequence was identified in the regulation of auxin metabolism in early SE [63]. Also, in a previous study using this species, miR827 was found expressed at high levels in a pooled sRNA library from samples including EC and developing somatic embryos [64]. Moreover, miR827 was predicted to target the *ETHYLENE-RESPONSIVE TRANSCRIPTION FACTOR 22* homolog in *N. benthamiana* [65], which in turn is involved in the SE regulation through modulation of ethylene and auxin responses in Arabidopsis [66]. Together, these results show the positive involvement of this miRNA in the embryogenic competence of several species.

In situ detection of miR827 confirms the successful application of FISH of sRNA in plant tissues [47], using conventional oligonucleotide probes [48]. Although in situ hybridization was previously employed to localize miR827 expression in rice, it was carried out with the resource of the expensive locked nucleic acid (LNA)-modified probes [67]. In that work, a strong signal was observed in tissues grown under Pi starvation, contrasting to a faint signal in sufficient Pi. This result revealed the accumulation of miR827 induced by low Pi levels. In *S. betaceum calli*, a stronger signal was detected in LTC compared to EC, corroborating the qPCR results and suggesting that LTC could be in a Pi-starvation-like condition (discussed below). The weak miR827 immunofluorescence signal observed in NEC may be attributed to the higher water content and large vacuoles of these cells [68], which make it difficult to fixate this kind of tissue and potentially hinder the binding of the antibodies. To our knowledge, localization of this miRNA has not been carried out in SE-related tissues.

Although no significant correlation was found between miR827 and its putative target gene (*PHT5)* expression, an opposite expression pattern was found in LTC and NEC. This result could suggest that miR827 has a role in the maintenance of the embryogenic potential of plant cell lines, through the regulation of P homeostasis, by negatively regulating *PHT5*.

To suppress the expression of the miRNAs in cells where they were upregulated (LTC), the VbMS approach was performed following the methodology described by Zhao et al. (2020) [69]. Besides not being significant, effective miRNA downregulation was achieved for miR827, since lower expression levels were quantified in transformed cells. Similarly, the STTM approach has been successfully used in the functional analysis of several miRNAs, in processes including embryogenesis, in a wide range of plant species [70]. In particular, the VbMS approach was applied in other solanaceous, such as *N. benthamiana* and *S. lycopersicum* [71]. The high efficiency of this method was confirmed using PVX for miR165/166 and miR159 silencing in *N. benthamiana*, in which highly reduced miRNA levels, higher levels of the target genes and phenotype modifications were observed [72]. This vector was also found efficient in *S. tuberosum* [69]. Although the downregulation of miR827 in LTC cells led to a decrease in the total P levels, in those cells, similar content was found for Pi.

Given that miR827 is described as induced in Pi-starvation conditions [24] and that this miRNA was found more expressed in cells with no embryogenic capacity, inorganic phosphate (Pi) and total phosphate (P) were quantified in EC, LTC and NEC, to assess whether this upregulation would be related with P contents. Indeed, much lower Pi and total P content were found in cells with no embryogenic competence (LTC and NEC). This result might explain the upregulation of miR827 in those cells, contrasting with the EC that revealed concomitant lower miRNA expression and higher P content. Considering Pi as a morphogenic elicitor, required for the acquisition of the embryogenic commitment [25], and that higher P levels are known to favour embryogenic and morphogenic responses [26–28] this result was expected. However, the mechanisms underlying the role of the Pi metabolism in embryogenic competence are far from fully understood. Several studies have suggested that changes in P availability influence auxin sensitivity [30, 31], ultimately related to embryogenic competence. Sánchez-Calderón and colleagues (2005) found that low P levels could be associated with the progressive loss of meristematic cells in the primary root [73]. Accordingly, as in root development, low levels of Pi in embryogenesis-related tissues can be hypothesized as leading to auxin hypersensitivity, which stimulates cell division at the expense of differentiation. In that sense, and given that EC was enriched in Pi, the effect of Pi concentration was analysed on SE induction and somatic embryo development. However, increasing the Pi availability on media did not result either in higher embryogenic competence acquisition or expression. This result indicates that the Pi concentrations tested (0, 0.6, 1.25, 2.5 and 5 mM, in SE induction, and 12.5 mM, in somatic embryo development) do not affect cell dedifferentiation or somatic embryo development in *S. betaceum*. Nevertheless, higher Pi concentration, that may indeed yield more pronounced effects, should be tested, as did Elkonin and Pakhomova (2000) [26] in sorghum. By increasing Pi levels up to 8.8–14.7 mM, EC induction and proliferation were promoted in that species [26]. Also, in *Cymbopogon schoenanthus*, the number of somatic embryos formed was higher at 62 mM Pi while the highest number of mature somatic embryos was achieved at 24.8 mM Pi [27]. Indeed, by the analysis of the composition of the zygotic embryos of olives, high phosphorus levels were found at the beginning of the embryo development, as for all mineral elements, but also at the maturation stage, similar to Na and contrasting with the low levels found for the other elements [74]. Shoot organogenesis from root explants is also improved in higher Pi levels. For instance, incubation of *Hippophae rhamnoides* seedlings in ten-fold higher Pi (12.5 mM) resulted in a higher number of shoots formed per root explant used [28]. Regarding morphogenesis, it was found that Arabidopsis plants grown at 1 µM P showed reduced growth of primary root than plants grown at 1 mM P levels [73]. However, 1 mM P is still lower than the concentration usually used to induce SE. Even though, the inhibition of primary root growth observed under Pi-deficient conditions and that also occurs by exogenous application of auxin [75] suggests that the continuous exogenous application of auxin during EC subcultures could be inhibiting the embryogenic potential through P-starvation mechanisms.

Sucrose has been pointed out as the mediator of Pi-starvation responses in plants [32–34]. Given the different Pi concentrations found among *S. betaceum* cell lines, sucrose was also quantified. However, no differences were revealed in the sucrose concentration in the different *calli*, using the anthrone quantification method. Although reliable and widely used for carbohydrate analysis, this method does not distinguish between sucrose and other sugars, such as glucose, fructose and starch, which may lead to an overestimation of the sucrose content in samples [76]. Other quantification methods should also be implemented, such as high-performance liquid chromatography (HPLC) or enzymatic methods, which allow the quantification of individual sugar components and, specifically, sucrose measurement [77]. The effect of sucrose availability on the medium was also analysed on the expression of the embryogenic competence. Those assays revealed that the high sucrose levels, in which *S. betaceum calli* is induced and proliferated, are required for cell proliferation. Furthermore, somatic embryo development occurred at a higher rate in high sucrose levels (0.26 M), suggesting that sucrose also promotes the expression of embryogenic competence. Nevertheless, in these conditions, the somatic embryos developed were not able to convert as occurs in lower sucrose levels (0.087 M). Thus, high sucrose levels promote somatic embryo development initiation, but subsequent incubation in lower levels is required for embryo germination and embling formation. Similar results were found during olive zygotic embryogenesis, in which high sucrose content was observed at the first stages and a marked decrease was found at the maturation phase [74]. Moreover, analysis of carbohydrate metabolism during spruce somatic embryo development revealed the importance of exogenous sucrose in SE efficiency [78]. Therefore, accurate sugar content in the culture medium would increase the efficiency of SE in woody species.

## Conclusions

Aiming to unravel the loss of embryogenic competence in *S. betaceum*, several assays for the functional characterization of miR827 were carried out and the involvement of the P homeostasis and sucrose accumulation was analysed. This miRNA was associated with lower P levels and with the loss of the embryogenic potential. However, to fully functional characterize this mechanism more research is required, including putative target validation and better modulation of Pi and sugar concentration in the induction and maintenance media. Nevertheless, altogether, these results supported the hypothesis that P metabolism is a determinant of embryogenic competence commitment, expression and maintenance during SE. More specifically, the present results suggest the need to maintain the high phosphate levels during long-term EC proliferation. Moreover, the present study suggests that miRNA-mediated gene expression regulation is involved in cell fate determination, including in the maintenance of embryogenic potential.

Overall, the present work paves the way for further functional characterization of the mechanisms underlying embryogenic competence in non-sequenced woody species, given the improvement of the techniques and protocols used. Also, attention should be paid to the potential biotechnological applications of these findings for the long-term conservation of EC cultures without the loss of embryogenic potential, particularly in economically relevant crops, and for more efficient induction of SE mainly in recalcitrant species.

## Electronic supplementary material

Below is the link to the electronic supplementary material.

Supplementary Material 1

## Data Availability

No datasets were generated or analysed during the current study.
