# Supplementary material for: Analysing the loss of embryogenic competence in long-term cell lines of Solanum betaceum Cav.: involvement of miR827, phosphate and sugar
Source: BMC Plant Biol. 2025 Jul 3;25:851. doi: 10.1186/s12870-025-06786-2 (PMC12224387; doi:10.1186/s12870-025-06786-2)
Supplement: Supplementary file 1 — Supplementary Material 1 [file 12870_2025_6786_MOESM1_ESM.pdf]

Analysing the loss of embryogenic competence in long-term cell lines of *Solanum betaceum* Cav.: involvement of miR827, phosphate and sugar

Daniela Cordeiro[1,2], Jorge Canhoto[1] and Sandra Correia[1,3*]

[1]Centre for Functional Ecology, Laboratory Associate TERRA, Department of Life Sciences, University of Coimbra, Calçada Martim de Freitas, 3000-456 Coimbra, Portugal

[2]Current address: UCIBIO – Applied Molecular Biosciences Unit, Department of Chemistry, NOVA School of Science and Technology, Universidade NOVA de Lisboa, 2829-516 Caparica, Portugal

[3]InnovPlantProtect CoLab, Estrada de Gil Vaz, 7350-478 Elvas, Portugal

[*]Corresponding author: Sandra Correia (sandraimc@uc.pt)

Table S1 The primers sequences and melting temperatures

Gene	Primer sequences (5'–3')	Tm (ºC)
**miR827 mature**	tt RT: TCAACAAATCAAGCTCTCCAGGTACAGTTGGTACCTGACTCCACGCGAATAG	68.5
	F: GCGGTCAACAAATCAAGCTC	54.8
	R: CCGCGTTTGTTGATGGTCAT	56.3
**miR827 star**	tt RT: GTTCATCTCTATCCTCTCCAGGTACAGTTGGTACCTGTCTCCACTTTGTTTG	66.8
	F: GCGTTCATCTCTATCCTCTCCA	55.8
	R: CGGCGTTAGATGAACATCAACAA	55.8
**miR166a mature**	tt RT: GAAGCCTGGTAAGAGAGTCCTCGTAGAGTTGCTACGAGATATGAATAGGGGAA	67.4
	F: GAAGCCTGGTAAGAGAGTCC	54.5
	R: GTCGGACCAGGCTTCATTC	55.8
PHT5	F: CGTCAGCCAGCCAACTCTAT	57.1
	R: CCAGCTTCGGTAGACAAGAGA	56.2
snoR14	F: TCATAAGTCTGTCAATCCACTGAA	53.7
	R: ACGATCAGAACCTGTCGGGA	58.0
UBQ10	F: GCAGCTCGAAGATGGACGTA	56.9
	R: AGTTCACAGCACGAGGATGG	57.3

Table S2 miRNA probes for *in situ* hybridization

miRNA	Probe sequence (5' → 3')	Tm (ºC)	Hybridization temperature (ºC)
**miR827**	TGTTTGTTGATGTTCATCTAA	52	54
**miR166a**	GGGGAATGAAGCCTGGTCCGA	62	56
**miR166a –**	TCGGACCAGGCTTCATTCCCC	65	56
**U6**	TCTCGATTTGTGCGTGTCAT	57	54
